# Effects of Fluctuating Daily Temperatures at Critical Thermal Extremes on *Aedes aegypti* Life-History Traits

**DOI:** 10.1371/journal.pone.0058824

**Published:** 2013-03-08

**Authors:** Lauren B. Carrington, M. Veronica Armijos, Louis Lambrechts, Christopher M. Barker, Thomas W. Scott

**Affiliations:** 1 Department of Entomology, University of California Davis, Davis, California, United States of America; 2 Center for Vectorborne Diseases, University of California Davis, Davis, California, United States of America; 3 Insects and Infectious Diseases, CNRS URA 3012, Institut Pasteur, Paris, France; 4 Department of Pathology, Microbiology and Immunology, University of California Davis, Davis, California, United States of America; 5 Fogarty International Center, National Institutes of Health, Bethesda, Maryland, United States of America; University of Texas Medical Branch, United States of America

## Abstract

**Background:**

The effect of temperature on insect biology is well understood under constant temperature conditions, but less so under more natural, fluctuating conditions. A fluctuating temperature profile around a mean of 26°C can alter *Aedes aegypti* vector competence for dengue viruses as well as numerous life-history traits, however, the effect of fluctuations on mosquitoes at critical thermal limits is unknown.

**Methodology/Principal Findings:**

We investigated the effects of large and small daily temperature fluctuations at low (16°C) and high (35–37°C) mean temperatures, after we identified these temperatures as being thresholds for immature development and/or adult reproduction under constant temperature conditions. We found that temperature effects on larval development time, larval survival and adult reproduction depend on the combination of mean temperature and magnitude of fluctuations. Importantly, observed degree-day estimates for mosquito development under fluctuating temperature profiles depart significantly (around 10–20%) from that predicted by constant temperatures of the same mean. At low mean temperatures, fluctuations reduce the thermal energy required to reach pupation relative to constant temperature, whereas at high mean temperatures additional thermal energy is required to complete development. A stage-structured model based on these empirical data predicts that fluctuations can significantly affect the intrinsic growth rate of mosquito populations.

**Conclusions/Significance:**

Our results indicate that by using constant temperatures, one could under- or over-estimate values for numerous life-history traits compared to more natural field conditions dependent upon the mean temperature. This complexity may in turn reduce the accuracy of population dynamics modeling and downstream applications for mosquito surveillance and disease prevention.

## Introduction

The effects of constant temperatures on the life-history traits of *Aedes aegypti* are well established [1]–[7]. Mosquitoes in the wild, however, are not exposed to constant temperatures as they are faced with temperature variation on a daily basis. Aquatic immature mosquitoes are subject to daily fluctuations in water temperature during development and adults experience similar changes in air temperature.

Numerous estimates and predictions of the effects of temperature on the stability of mosquito populations, expansion or contraction of geographic range, and vector competence have been published [8]–[12]. Each of these studies is based on estimates made under constant temperatures, to which mosquitoes are not naturally exposed. Whether mosquitoes live indoors, outdoors, or occupy a composite thermal environment by moving in and out of sheltered habitats, mosquitoes experience some degree of temperature variation throughout the day [13], [14].

Recent studies have shown that diurnal temperature range (DTR) can alter estimates of immature development in Anopheline and Aedine mosquitoes and their vector competence for malaria parasites and dengue viruses (DENV) [15]–[17]. The magnitude of fluctuations, as well as the mean around which they oscillate, are likely to alter the direction of the effect of DTR on these traits. Specifically, a reduction in *Ae. aegypti* vector competence for flaviviruses is predicted when fluctuations occur at mean temperatures above 18°C, whereas transmission is expected to increase when fluctuations occur at mean temperatures below 18°C [16]. Empirical studies confirm that a large DTR (∼20°C) reduces *Ae. aegypti* midgut infection rates around an intermediate mean of 26°C [16], [18]. With regard to life-history traits, a large DTR at the same mean reduces female fecundity, larval survival and extends development time of *Ae. aegypti*. A small DTR (∼8°C) on the other hand, accelerates the speed of development of males, and increases female reproduction, although the effects are less discernable [17]. In comparisons between field and laboratory reared mosquitoes, Tun-Lin et al. [4] found that development in the field is generally much slower than under laboratory conditions, and containers with smaller volumes of water had greater variation in temperature than containers with larger water volumes. Although it remains unclear from this study to what extent temperature and DTR interact to alter mosquito development, the results suggest a complex interplay between the two variables. Conversely, Richardson et al. [7] found that constant temperatures could be used to accurately predict the development of Australian *Ae. aegypti* reared under fluctuating temperatures, but specific rearing temperatures were not described.

Such responses to temperature variation are not restricted to Aedine mosquitoes. Paaijmans et al. [19] found that fluctuations around both high and low mean temperatures altered development and survival rates of *Anopheles stephensi*. A DTR of 12°C around a mean of 27°C reduced survival, extended development time, and slowed malaria development, while the same DTR at 20°C had the opposite effect.

Current knowledge on the effects of realistic temperature fluctuations on *Ae. aegypti* biology is limited to results recently reported for variation around a mean of 26°C [17]. Given the potential for changes in both vector competence and life-history traits when temperatures fluctuate around different means, something that could lead to altered population dynamics, we characterized responses by a population of *Ae. aegypti* from Kamphaeng Phet in Thailand to determine the minimum and maximum temperatures at which this species can develop and reproduce. We then explored the effect of small and large fluctuations at those low and high mean temperatures and measured responses relative to mosquitoes under a constant, control temperature. Data was then used to calculate the required number of degree-days (DDs) for larval development for this mosquito population, and we tested whether daily fluctuations would affect an estimated development rate. Finally, we created a dynamic model that establishes expectations for the thermal limits under which this population may persist based on our empirical immature and adult data sets. Our results indicate that both large and small fluctuations in temperature have the potential to impact life-history traits and alter *Ae. aegypti* population dynamics.

## Methods

### Experimental Design

We tested the effect of temperature on a number of life-history traits of *Ae. aegypti* over a series of six experiments. Each experiment assessed larval development time and survival, and a number of female reproductive traits, including the pre-blood meal period, length of the gonotrophic cycle, and the number of eggs in the first gonotrophic cycle (assays are described in detail below). Temperatures tested ranged between 12°C and 40°C, with 4–6°C intervals. After first identifying upper and lower thermal limits of development and reproduction under constant temperatures, we then explored the effect of diurnal fluctuations around these minimum and maximum temperatures. Low temperatures were tested together (Experiment 1∶12°C, 16°C and 20°C), as were higher temperatures (Experiment 2∶30°C, 35°C and 40°C). Between the temperatures of 35°C and 40°C, we needed greater resolution to identify the upper survival threshold for this population, so we tested 39°C (Experiment 3), and 37°C and 38°C (Experiment 4). Finally, we tested the effect of diurnal fluctuations around low (16°C) and high (35–37°C) mean temperatures (Experiments 5 and 6, respectively), with the constant temperature re-tested as an internal experimental control.

The amplitudes of the fluctuations used in the experiments were either *small* (7.6°C) or *large* (18.6°C). These profiles represent the shape and magnitude of DTRs observed in the high and low DENV transmission seasons in central west Thailand. The profiles followed a truncated sinusoidal progression during the day and exponential decrease at night, because this more accurately represents the profile of daily temperature variation compared to a symmetrical sinusoidal profile [16], [20]. The shapes of the profiles were the same for each of the fluctuating temperature experiments, but the profile was raised or lowered in order to adjust the mean temperature as necessary. Temperatures were maintained in Binder KBF115 environmental chambers (Tuttlingen, Germany). Data loggers, both internal in the chamber and independent HOBO data loggers (Onset, Cape Cod, MA), recorded temperature and humidity variation. Actual temperatures were within 0.3°C of the programmed temperatures throughout the duration of the experiments. The relative humidity level was maintained between 70% and 80% across all experiments.

Using this empirical development time data, we calculated the required DDs for *Ae. aegypti* larval development, and constructed a stage-structured model to estimate the impacts of the temperature regimes on *Ae. aegypti* population dynamics. We also re-analyzed data from Carrington et al. [17], which were generated using the same laboratory protocols, to include in the latter portion of this study. In this report, we make the distinction between the effect of temperature and DTR. We use temperature to refer to changes in mean constant temperatures (e.g.; 16°C, 20°C, 30°C etc) and DTR to refer to differences between the magnitudes of fluctuations around a given mean temperature (e.g.; *constant* [where the DTR = 0°C], *small* DTR or *large* DTR).

### Mosquitoes

We used *Ae. aegypti* collected from Kamphaeng Phet (KPP), Thailand in our experiments. Originally collected from the field in January 2011, the mosquitoes were maintained under standard laboratory conditions until they were used for experiments. Eggs were hatched in water using a vacuum manifold and reared under a controlled density (≈200 larvae per tray) in containers (≈33×29×6 cm) with 1.5L of deionized water. Colonies were maintained with a population size of >500 individuals per generation. Larvae were fed as previously described in Styer et al. [21]. The F_4_ generation of mosquitoes was assessed in our life-history trait assays.

### Development Time and Survival

For each experiment, estimates of egg to pupa development time were made by rearing mosquitoes in replicate rearing cups, with 20 individuals per cup with 100 ml deionized water, hatched under vacuum to ensure minimal variation of hatching time. We tested between 13 and 20 replicate cups per temperature treatment. For each temperature, pre-warmed water was used to replenish the evaporated water every day at the same time that food was administered. We assessed survival daily (except for the first day, due to the fragility of the newly-hatched first instar larvae) by counting the number of remaining larvae left in each cup. Mean development time from egg hatch to pupation was recorded in hours or days. High temperature experiments (a mean of 26° and above) were scored every 6 hours from first pupation to the last and low temperature experiments (≤20°C) were scored once daily due to slower development rates. Median time from pupation to emergence was recorded to enable complete population dynamic estimates for subsequent modeling.

Based on a linear regression constructed from the rates of development for each rearing cup at constant temperatures between 16°C and 35°C, we calculated the minimum development threshold temperature, and subsequently determined the DD required for the KPP population *Ae. aegypti* to pupate.

### Egg Production

Mosquitoes assessed in experiments were all reared under the temperatures at which they were tested, with controlled densities in experimental cages. Between 10 and 18 females reared per temperature were each placed with a single male within 24 hours of emergence. We measured the length of time from emergence until the first blood meal (termed the pre-blood meal period), the time it took from the first blood meal to the first day eggs were observed (length of the first gonotrophic cycle) and the number of eggs that were laid (clutch size). Human blood (L.B.C.) was offered for 15 minutes every day from the start of the experiment, until two days after the first blood meal of each female, which allowed females two subsequent opportunities to feed to repletion. If a female had still not fed on the third day after the experiment began (or fifth day at the lower temperatures), she was removed from the analysis. Females at higher temperatures were allowed a minimum of seven days to lay eggs. At the cooler temperature treatments females were allowed 3 weeks.

The University of California at Davis Institutional Review Board determined that this experiment (allowing mosquitoes to take blood meals from people) did not meet the criteria for human subjects research and thus, did not require human subjects approval.

### Statistical Analysis

#### Development time

All analyses were conducted using JMP (SAS Inc., NC, USA). For immature traits, we used a Kaplan-Meier analysis (Log-rank tests) to assess the effect of temperature on development time and survival. We additionally used a multivariate Analysis of Variance (MANOVA) to determine the effect of temperature on mean development time (hatching to pupation) and percentage of larvae surviving to pupation for each cup. We then used univariate Analyses of Variance (ANOVA) as post-hoc tests to determine which individual traits were affected by these factors. Each test was performed both with and without the 26°C treatment from Carrington et al. [17], with no difference in the level of significance between tests observed. Statistical results presented include the 26°C temperature treatment. ANOVA was also applied to estimates of the required DDs for each replicate rearing cup.

#### Reproduction

We applied a MANOVA to fecundity data (pre-blood meal period, length of the gonotrophic cycle and clutch size) to assess the effect of temperature in initial experiments and DTR in subsequent experiments on each female. ANOVAs were then used to determine which particular traits tested within the MANOVA were actually affected. As for the immature data, we included the 26°C data from Carrington et al. [17] and found no differences among test results.

### Modeling

We constructed a simple stage-structure matrix model [22] for *Ae. aegypti* population dynamics to allow us to translate experimental differences in life-history parameters into real-world expectations for potential population growth in the absence of other limiting influences such as density dependence, predation or inter-specific competition. This allowed us to identify the temperatures and DTRs that maximize reproductive potential for *Ae. aegypti*. The model, written in R version 2.15 [23] used median development times to pupation and adult emergence, and fecundity estimates for adult females at each temperature. Parameter estimates used in the model were based on estimates taken directly from our KPP population in this study. Remaining estimates, for those traits we did not measure, were based on the following published measures and assumptions. Because *Ae. aegypti* eggs can maintain viability for up to one year [24], we standardized hatch time between treatment groups to five days, with a 99% chance of survival each day. We assume the female:male ratio is 1∶1 for offspring, and that mating did not limit population dynamics. Based on our initial measurements of components of the gonotrophic cycle at 26°C constant temperature [17], we reduced the clutch size by 5% each subsequent cycle after the first, and extended the duration of each gonotrophic cycle by 15% [17], [25]. A 90% survival rate per day was assumed for adult females [25], [26]. Because we had not observed females to complete more than four gonotrophic cycles in our previous study with this population at 26°C [17], we assumed that females die after completion of this fourth cycle. Temperature-DTR combinations that did not result in viable offspring in our experiments (e.g., if females did not lay eggs or immatures did not survive to adult emergence) were not considered for modeling.

## Results

We first describe the results from the immature life-history traits (larval development time and larval/pupal survival) and DD calculations. This is followed by presentation of adult traits (length of pre-blood meal period, length of the first gonotrophic cycle, and the number of eggs in the first gonotrophic cycle). Within each section, we describe the combined results from experiments at constant temperatures first and then those with fluctuations. Finally, the predictions of the model are presented.

### Immature Traits

#### Constant temperatures

**Figure 1:**
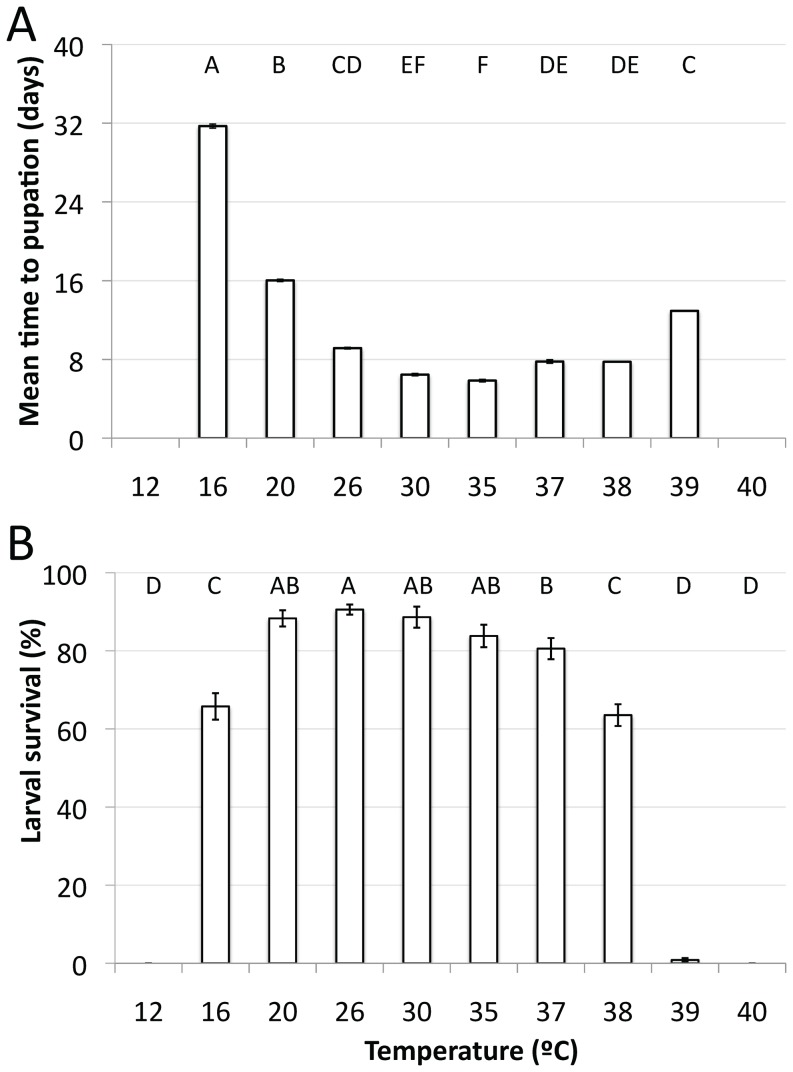
Development time and survival estimates for *Ae. aegypti* at a range of constant temperatures. (A) Mean development time in days from egg hatching to pupation and (B) Mean percentage of larval survival across temperature ranges. Treatments with the same letter above each bar are not statistically different from each other according to a Tukey HSD post-hoc test. Note that 26°C was performed in the study by Carrington et al. [17], but has been included here for comparative purposes.

Larval density can influence development time, therefore we considered the effect of temperature jointly on development time and survival estimates using a multivariate analysis of variance (MANOVA). Temperature significantly influenced development time and survival (Wilk’s Λ_14, 186_ = 168.105, p<0.0001). Ancillary ANOVAs on these individual traits indicated that temperature impacted both traits (development time: F_7, 94_ = 612.02, p<0.0001; survival: F_9, 135_ = 364.87, p<0.0001). Development time was fastest at 35°C (mean development time 5.85 days ±0.133 SE), and slowest at 16°C (31.7 days ±0.48 SE). At this low temperature, the single longest time it took an individual to pupate was 92 days (it died shortly after pupation). As this individual was clearly an outlier (by more than 1 month), we excluded this individual from the analysis. None of the larvae survived until pupation at 12°C and 40°C, and as such, we were not able to obtain an estimate of development time for those treatments. Mean times until death, however, were 7.44 and 2.67 days, respectively. Less than 1% larval survival was observed for 39°C. A total of three larvae pupated at this temperature, although they were already dead at the time they were first observed. At 16°C and 38°C larval survival was also low (65.7% and 63.5% survival respectively). The highest larval survival was seen at 26°C (90.5%), closely followed by 30°C (88.6%) and 20°C (88.3%). Survival rates for pupae ranged between >80% for all temperatures between 20°C and 37°C. At 38°C only 11% of pupae survived to become adults, and they were small, feeble and inactive. Mean larval survival and development times are shown in Figure 1 (error bars indicate ±1 standard error of the mean).

**Figure 2:**
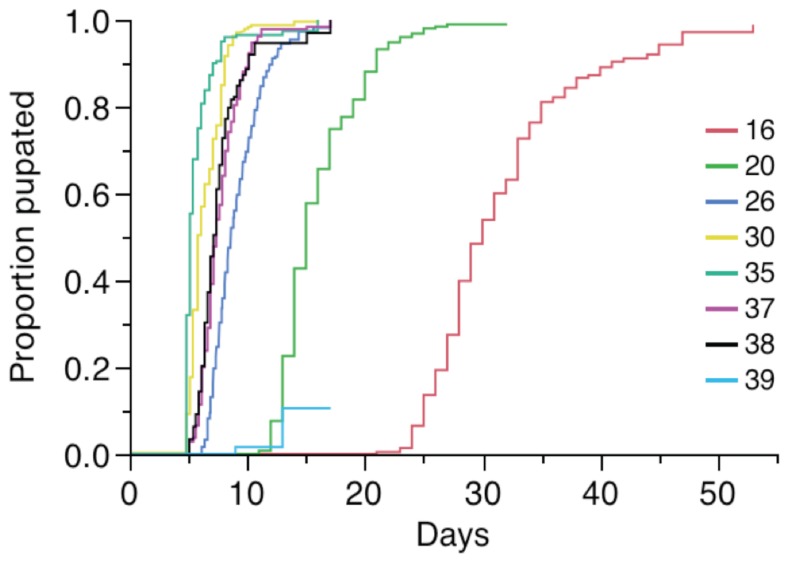
Development curves for *Ae. aegypti* mosquitoes reared under constant temperatures ranging from 16°C to 39°C. Each color represents a temperatures treatment, indicated at the right. There was a slightly significant effect of temperature on development time (χ^2^ = 2551.02, df = 8, p<0.0001). Note that 26°C was performed in the study by Carrington et al. [17], but has been included here for comparative purposes.

According to log-rank tests, temperature influenced larval development curves (χ^2^ = 2890.53, df = 9, p<0.0001), consistent with the results above. All temperature treatments were statistically different from each other in pair-wise analyses, except between the 20°C and 39°C treatments (χ^2^ = 2.11, df = 1, p = 0.146) and the 37°C and 38°C treatments (χ^2^ = 0.469, df = 1, p = 0.493). Figure 2 shows the development curves for each of the temperatures at which we successfully obtained pupae (between 16°C and 39°C).

#### Low temperatures with fluctuations

**Figure 3:**
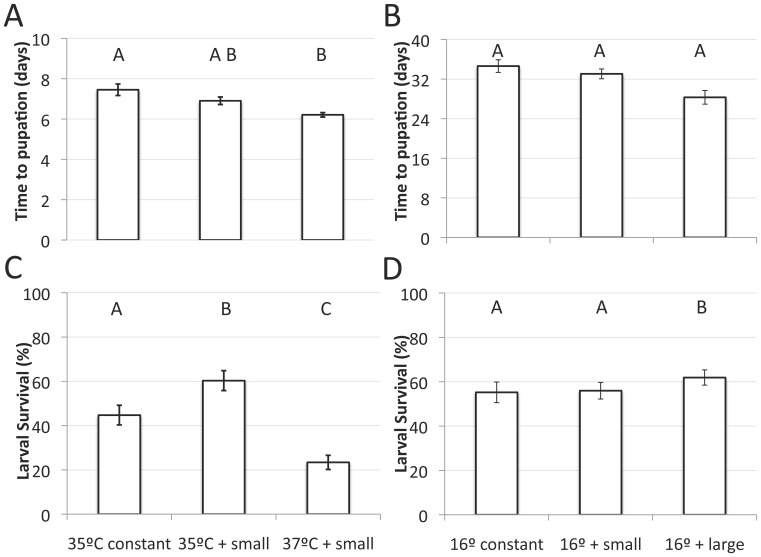
Effects of fluctuations around high and low mean temperatures on immature development time and survival. (A and B) Estimates of development time for the high and low temperatures, respectively. (C and D) Percentage larval survival for the high and low temperature experiments, respectively. Error bars represent ±1 standard error of the mean. Treatments with the same letter above each bar are not statistically different from each other according to a Tukey HSD post-hoc test.

MANOVA indicated that DTR influenced larval development time and survival at 16°C (Wilk’s Λ_4, 82_ = 4.781, p = 0.0019). ANOVAs demonstrate an effect of DTR on development time (F_2, 42_ = 7.23, p = 0.002; Figure 3B), but not larval survival (F_2, 42_ = 0.84, p = 0.437; Figure 3D).

Log-rank tests confirm the effect of DTR on development rates at 16°C (χ^2^ = 44.45, df = 2, p<0.0001). The large DTR significantly reduced mean development time (30.7 days) compared to both the constant temperature (35.7 days; χ^2^ = 33.91, df = 1, p<0.0001) and small fluctuation (34.7 days; χ^2^ = 24.95, df = 1, p<0.0001). There was no difference between the small DTR and constant temperature regimes (χ^2^ = 0.714, df = 1, p = 0.397).

#### High temperatures with fluctuations

Temperatures beyond 37°C significantly lowered the survival of immature mosquitoes, and beyond 35°C there was a reduction in the ability of females to reproduce (see below). We therefore tested mosquitoes at a constant temperature of 35°C and two temperature regimes with small diurnal fluctuations (35°C and 37°C), representing the thresholds for development and reproduction, which each temperature regime treated as a nominal variable.

Temperature treatment significantly altered mosquito development time and survival according to MANOVA (Wilk’s Λ_2, 42_ = 13.393, p<0.0001). Development time and survival were both significantly affected by temperature treatments (F_2, 42_ = 9.394, p = 0.0004 and F_2, 42_ = 20.831, p<0.0001 respectively). Egg-to-pupae development time increased from 6.11 days at 35°C constant to 6.50 days with a small fluctuation at the same mean. Development time under small fluctuations at 37°C rose to 7.37 days. Adults emerging from the 37°C+small DTR treatment responded very similarly to those reared at a constant 38°C, in that they were inactive, weak, and died shortly after emergence. Only the 35°C constant and 37°C small DTR treatment were different from each other in mean development times (Figure 3A). Larval survival increased from a baseline of 45% under a constant 35°C (our internal control treatment) to 60% with the addition of small fluctuations, but dropped to 23% at 37°C with small fluctuations. Survival rates of all treatments were significantly different from each other (Figure 3C). Pupal survival remained high when the mean temperature was 35°C, irrespective of DTR (>95%), but with a small increase in mean temperature to 37°C, pupal survival dropped to 51%.

Log-rank tests confirmed the overall effect of temperature on differing development curves at high temperatures (χ^2^ = 40.44, df = 2, p<0.0001), and pair-wise comparisons demonstrated that each curve was different from the other two treatments (p<0.005 in each test).

#### Degree-day calculations

The minimum temperature threshold for development of the KPP population was estimated at 11.78°C based on the linear regression between 16°C and 35°C (R^2^ = 0.982). Based on this minimum threshold, we calculated that mosquitoes required an average of 126.38 (±1.49 SE) DD for larval development. We observed no difference between the calculated DDs for each of the five constant temperature regimes based on the means of each replicate cup (F_4, 69_ = 1.458, p = 0.224).

**Figure 4:**
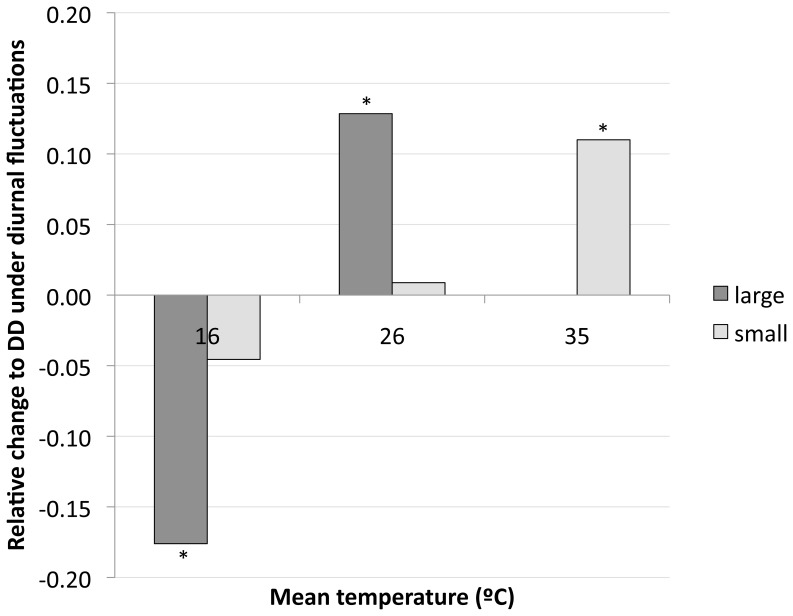
Relative changes in the degree-days required for larval development under constant and fluctuating temperatures. Zero on the y-axis represents the estimated degree-days for constant temperature development, with the bars in either direction indicating the relative change in degree-days needed by mosquitoes under respective fluctuating temperature treatments to reach pupation. Asterisks indicate that the change is significantly different to the constant temperature control (p<0.01).

The addition of fluctuations did result in differences in degree-day requirements. For a 16°C mean temperature, effects of DTR on DD estimates were significant (F_2, 39_ = 5.47, p = 0.008). A small DTR reduced DDs by ∼4%, while a large DTR reduced DDs by 18%. DTR also influenced DD estimates around an intermediate mean of 26°C (F_2, 45_ = 8.02, p = 0.001), but in the opposite direction. There was a negligible difference between the small and constant temperature regimes (<1%), but 13% more DDs were required for mosquitoes to develop under the large DTR compared to constant temperatures. At the upper end of the scale, small fluctuations around a 35°C mean produced an 11% increase in DDs required to reach pupation (F_1, 28_ = 10.64, p = 0.002). We did not test the 37°C+small DTR treatment because 37°C was out of the range for linear development for this population. Figure 4 depicts these relative changes compared to constant temperatures.

### Adult Reproductive Traits

#### Constant temperatures

**Figure 5:**
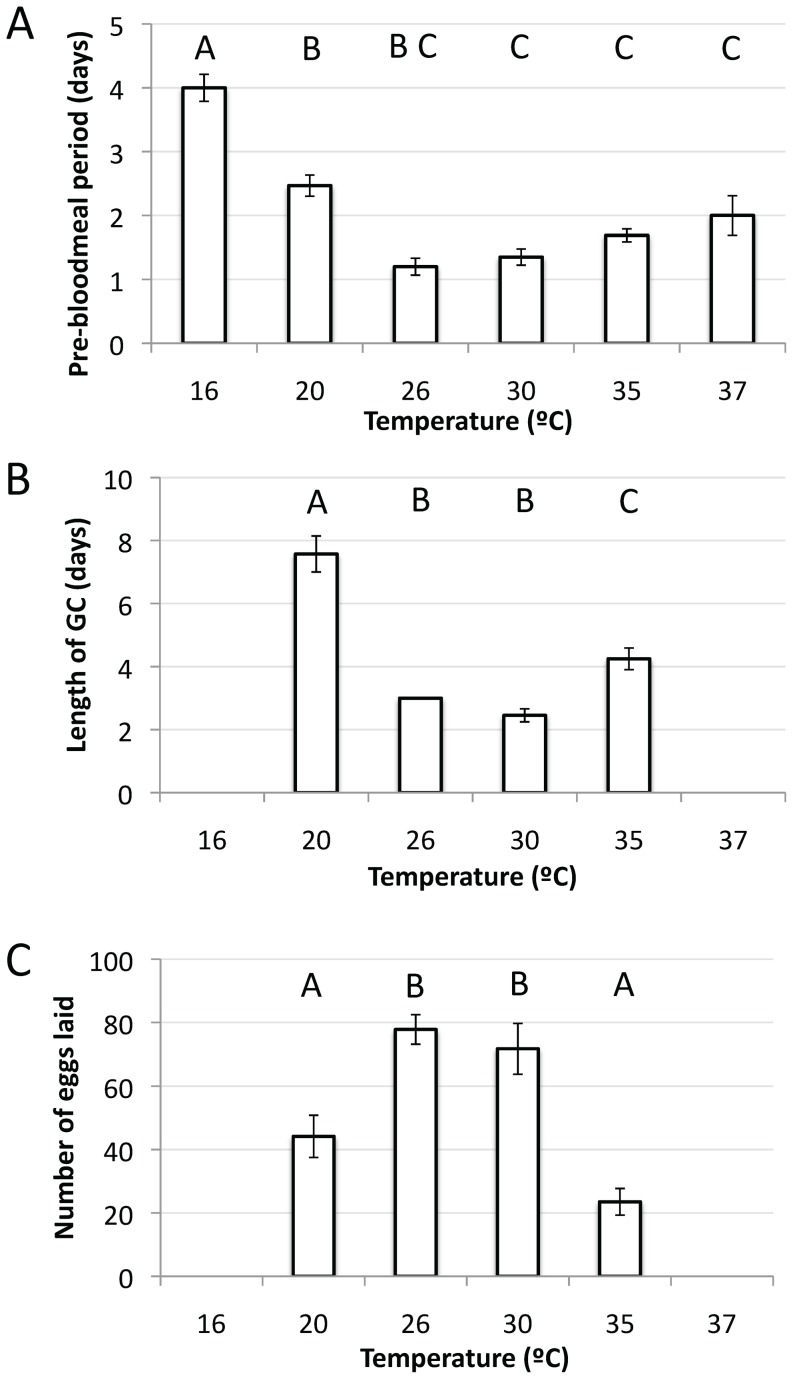
Summary of the effects of six constant temperature treatments on three female reproductive traits. (A) Mean number of days before a female took a blood meal from a human; (B) Mean length of the gonotrophic cycle (measured from the time of the first blood meal to the time the first eggs were laid); and (C) Mean number of eggs paid per female in their first gonotrophic cycle. Error bars represent ±1 standard error of the mean. Missing values in panels B and C for the 16°C and 37°C treatments are because no eggs were laid by any female. Treatments with the same letter above each bar are not statistically different from each other according to a Tukey HSD post-hoc test. Note that 26°C was performed in the study by Carrington et al. [17], but has been included here for comparative purposes.

Ninety-eight females were assessed for three reproductive traits (pre-blood meal period, length of gonotrophic cycle and clutch size) in the first experiments, testing the effect of constant temperature from 16°C up to 37°C. According to a MANOVA, temperature influenced *Ae. aegypti* reproductive traits (Wilk’s Λ_9, 73.16_ = 17.20, p<0.0001). Mosquitoes from all temperatures treatments fed on blood, except at 38°C. Temperature significantly influenced the timing of the pre-blood meal period; females reared at 30°C to 37°C took their first blood meal within 48 hours of emergence (F_5, 63_ = 35.71, p<0.0001), with females reared at 26°C having the shortest pre-blood meal period (Figure 5A). Eggs were produced by females from temperature regimes between 20°C to 35°C only, indicating that the range of temperatures tested encompassed both the minimum and maximum thresholds for egg production for this population. Accordingly, a significant effect of temperature was identified (F_3, 32_ = 16.98, p<0.0001). Peak egg laying was observed at 26°C, with >75 eggs laid per female in their first gonotrophic cycle (Figure 5C). The shortest gonotrophic cycle was also observed at this temperature (Figure 5B), which differed significantly from both the 20°C and 35°C treatments (F_3, 32_ = 48.12, p<0.0001).

#### Low temperatures with fluctuations

When both small and large fluctuations were added to a mean temperature of 16°C, the number of females feeding increased from 50% to up to 100%. Consistent with the previous result at the 16°C constant temperature regime, females that blood fed failed to lay eggs after more than three weeks. Despite the increase in DTR and greater number of females that fed, all females in both the small and large DTR regimes failed to lay eggs after an allowed 3-week oviposition period.

#### High temperatures with fluctuations

Females from the highest temperature treatment (37°C+small DTR) responded very similarly to mosquitoes from the 38°C constant temperature treatment in the first experiments; all died within the first days after emergence. We had no blood feeding success at this temperature. At 35°C with small fluctuations and despite having daily access, no females were interested in feeding on human blood. They showed no attempts to move toward the blood source. Females from the 35°C constant treatment were the only ones to successfully take a blood meal, which occurred an average of 2 days (±0.24 SE) after emergence. The mean number of eggs laid was 25.5 eggs/female (±7.71 SE), after 5 days (±0.58 SE).

### Modeling

Using empirical data from this study, our deterministic model allowed us to collectively evaluate the impacts of all life-table parameters of *Ae. aegypti.* Our experimental data showed that the KPP population may be sustained under constant temperatures ranging between 20°C and 35°C, but reproduction was not successful at 16°C (both with and without fluctuations) or at 35°C and 37°C with small fluctuations. As a result, population dynamics could not be modeled for these temperature treatments.

**Figure 6:**
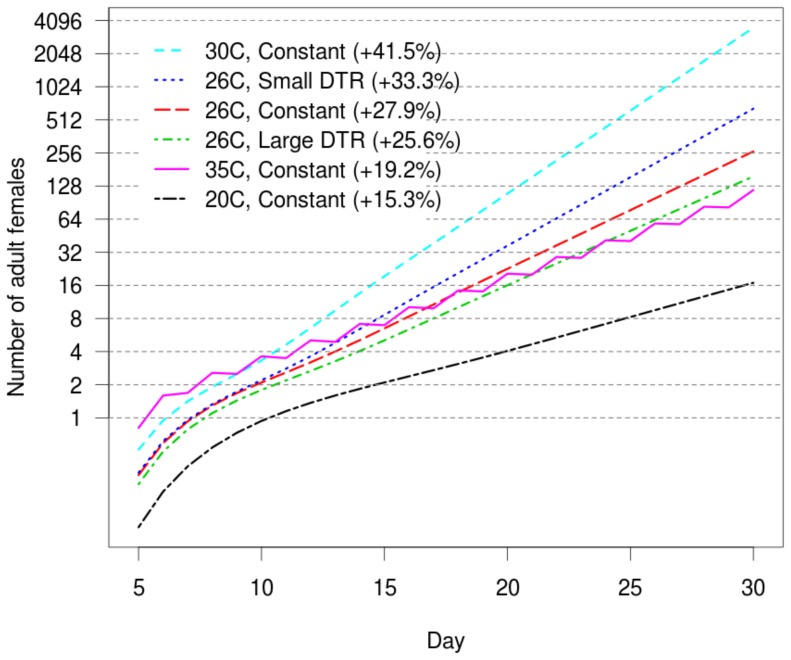
Predicted population growth of female *Ae. aegypti* after 30 days, starting with 10 viable eggs. Percentages for each temperature treatment indicate the projected exponential population growth rates per day. Note that the y-axis is on a log_2_ scale, so that each successive horizontal line represents a doubling of the population size.

A temperature of 26°C with both small and large fluctuations, however, sustained population growth and our model predicts that the previously reported ‘negligible or slightly positive’ effects on mosquito life-history traits observed under small fluctuations [17] would lead to a population size more than double that of the constant 26°C treatment after one month (Figure 6). Daily population growth rates were 33.3% and 27.9% respectively. Conversely, a large DTR at the same temperature results in a 40% reduction in the adult mosquito population size compared to the constant temperature control, resulting from a slightly lower growth rate of 25.6%. According to the model, the optimum temperature for population growth is 30°C. At this temperature, the model predicts a growth rate of 41.5% each day, leading to more than 60,000 eggs and 3,525 adults in the population after 30 days. The least optimal temperature for population growth (15.3% per day) is 20°C, with 16 adults after 30 days,

## Discussion

In this study we expanded upon our recent work [17], which characterised a population of *Ae. aegypti* from Kamphaeng Phet, Thailand for immature and adult life-history traits under small and large fluctuating temperature regimes around a mean of 26°C. Here, we examined various thermal profiles at mean temperatures higher and lower than 26°C. We identified the thermal minimum, optimum and maximum for larval development time and survival, blood feeding and reproduction at constant temperatures. Finally, we constructed a DD model for larval development and a population dynamic model predicting population growth rates under various constant and fluctuating temperature regimes. Our results show that under fluctuating conditions, estimates for these traits may be significantly altered compared to constant temperatures, with the direction dependent upon the mean temperature and the magnitude of the temperature fluctuations. The use of constant temperatures in the laboratory may not accurately reflect variable environmental conditions in the field; estimates of development time, degree-days, survival and reproductive ability can all be different under constant *vs.* more natural fluctuating temperatures.

An overall conclusion from our study is that due to the differences in mosquito life-history traits that we detected, better understanding of daily temperature variation on insect physiology will lead to improved vector control by local authorities and long-term management of vector-borne disease risk. It is important to note that in this context our results are specific to the mosquito population we studied and, although we do not expect the overall trends to vary significantly, detailed responses will likely vary among different *Ae. aegypti* populations.

### Immature Development Time and Survival

Development time estimates were highly temperature sensitive, and inversely related to temperature up to a maximum of 35°C. Beyond this threshold, the rate of development declined. The addition of a small DTR around 16°C and 35°C did not alter development time relative to the constant temperature control, but at 16°C a large DTR significantly accelerated development. A similar result demonstrating the importance of DTR magnitude was reported for a large DTR at 26°C [17]. Richardson et al. [7] report conflicting results to these. They reported that the development time of mosquitoes reared under constant temperatures accurately predicted development time of mosquitoes under fluctuating conditions. While the temperature profiles of their experimental fluctuating temperature regimes were not reported, our results suggest that DTR in their cyclical temperature treatments were similar to the magnitude of our ‘small DTR’ (i.e.; <10°C). Tun-Lin et al. [4] explored *Ae. aegypti* development directly under field conditions, in various sized containers and with different water volumes. They found that field-reared mosquitoes had greater variability in development rates compared to those reared in the laboratory, especially in smaller containers where water temperature fluctuated more extensively than in larger containers. Only 50% of comparisons between survival data from the laboratory and the field were similar, demonstrating that the DTR of water can influence immature mosquito development and survival. Other laboratory experiments have shown that fluctuating temperatures can modify the dependence of traits on temperature. *Ae. aegypti* from Trinidad reared at constant temperatures had a significant temperature-dependence of wing length, but under fluctuating water temperatures, this effect disappeared [6].

Although the mean temperature a mosquito is exposed to throughout the day may be the same across DTR treatments, as indicated by significant effects of DTR on our DD estimates, there appears to be an underlying physiological response of the mosquito to the daily variation in temperature that contributes to changes in the speed of development that is not observed under equivalent constant temperatures. Directionality of the change is dependent on mean temperature. At intermediate and high temperatures, both large and small fluctuations (at 26°C and 35°C respectively) resulted in mosquitoes needing a greater amount of thermal energy to reach pupation than under the constant temperature control. At low temperatures, however, large fluctuations significantly reduce this number of DDs, despite the exposure temperatures dropping below the minimum for development for portions of the day. We are unaware of any studies that have performed comparable experiments with realistic fluctuations in temperature in other species.

These results underline the importance of using appropriate temperature regimes when measuring population parameters in order to accurately describe mosquito life-history. Without considering this variation, it is possible that development rates of mosquitoes (and other insects) in nature are either under- or overestimated, dependent upon mean temperature.

### Fecundity

High mean temperatures with fluctuations were detrimental for mosquito reproduction. The addition of even a small DTR to a 35°C mean temperature terminated reproduction, presumably because the maximum temperature reached during the day (∼39.7°C) was damaging to the mosquitoes. Initial testing demonstrated that mosquitoes did not reproduce at 16°C, but we hypothesised that the addition of fluctuations might rescue female reproduction. Despite an increase in blood feeding activity, there were no eggs laid by any female exposed to fluctuating temperatures around of mean of 16°C, suggesting that either this mean temperature or the extremely low overnight temperatures are detrimental to reproduction.

It is possible that the females that did not lay eggs in these experiments were in fact unmated; we did not physically observe all females copulate and we did not dissect ovaries to confirm insemination and egg development in the blood fed females. Future experiments would benefit from allowing females more time to lay eggs, and subsequently dissecting individuals to determine stages of egg development. Another possibility is that these low temperatures negatively impacted male fertility. This issue needs further examination. Although we cannot offer an explanation to the cause of failed reproduction, this result is biologically meaningful. In our experiment, we reproduced field conditions by rearing both sexes and allowing mating to occur at the temperatures at which they were tested.

Unfortunately, we were unable to test for statistical significance between temperature-DTR treatments at high and low temperatures for reproduction because no estimates were obtained. These descriptive results, however, strongly suggest that DTR at both upper and lower ends of the temperature scale can alter estimates of life-history traits relative to constant temperatures. Predictions of mosquito responses to temperature in the wild based on laboratory estimates should thus be made with caution. Despite a lack of reproduction it is important to note that the continued blood feeding of mosquitoes at these high and low temperatures may still allow for the transmission of mosquito-borne pathogens, including DENV which can be transmitted at temperatures as low as 13°C [27].

Previous studies have shown that both temperature and humidity have the ability to alter fecundity estimates. Costa [5] considered small temperature fluctuations (DTR = 4°C) around each of three mean temperatures, with two humidity profiles. Fecundity was reduced with increasing mean temperatures, as was the length of the gonotrophic cycle. Increasing humidity also intensified the effects of temperature. Constant temperature controls were not included so the influence of fluctuations could not be teased apart.

### Modeling

Our model predicts population growth for *Ae. aegypti* exposed to various temperature-DTR combinations and demonstrates that constant temperatures around 30°C are optimal for the KPP population. Our simple model does not consider influences of inter-specific competition, limited access to nutrition or predation, and, therefore, our results inform on growth rate under optimal conditions. Under field conditions, each of these factors can influence the development and/or fecundity of mosquitoes and would be expected to limit population growth. The 26°C small and large DTR temperature treatments are representative of conditions observed in the high and low DENV transmission seasons in central west Thailand [17]. Relative to a constant 26°C, our model predicts that the potential for mosquito vector population growth under small daily fluctuations is markedly higher (+5.4% difference in daily population growth rates), whereas under large fluctuations, population growth is reduced (–2.3%). Entomological surveys in nearby areas support seasonal mosquito density changes [28] and these changes are positively associated with DENV transmission patterns.

Our population dynamic model could only be run for each temperature when we obtained mosquito trait values after they completed each stage in the model. The lack of experimental data we obtained at particular high and low temperature-DTR combinations, which is a result in itself, meant that we were unable to obtain output at those temperature regimes. We did not assess the effect of fluctuations at constant 30°C and constant 20°C, which the model ranked as the most and least optimal conditions for population growth, respectively.

Modeling of *Ae. aegypti* population dynamics under constant and cyclic temperatures indicates that more in depth studies are required to fully understand the complexities of environmental variation of life-history traits. Predictions of population growth and decline based on environmental temperature in the wild may be inaccurate due to differences between the mean temperatures observed and the DTR experienced by the mosquitoes. For example, our study population of KPP *Ae. aegypti* have a calculated thermal minimum for development of 11.78°C. We know, however, that development under a 16°C+large DTR regime still occurs, despite the fact that temperature drops ∼4°C below this thermal minimum for around 1/3 of the day. In fact, development occurs at a rate faster than that of mosquitoes reared at a 16°C constant temperature regime. It might be expected that the time to development would be longer for a mosquito experiencing sub-optimal temperatures, given that a portion of the day is spent below the thermal minimum for development, but in fact it takes less time and fewer degree-days to develop. The thermal minimum for development, therefore, must be interpreted cautiously.

### Conclusions

Fluctuating temperatures alter estimates of life-history traits in immature and adult *Ae. aegypti* with the direction of the change dependent upon the magnitude of the DTR and mean temperature around which the fluctuations oscillate. Differences in DD estimates between fluctuating and constant temperature treatments of the same mean reveal a physiological response by the mosquitoes that modify their normal developmental rate. Results from this study demonstrate the sensitivity of life-history traits to temperature fluctuations at the upper and lower thermal limits and suggests that standard constant temperature laboratory-based experiments do not fully reflect what is happening in nature. Vector control efforts and population dynamic models will be improved when realistic parameter estimates of mosquito populations are incorporated in future surveillance activities and research projects.
